# Ripple effects mapping: capturing the wider impacts of systems change efforts in public health

**DOI:** 10.1186/s12874-022-01570-4

**Published:** 2022-03-18

**Authors:** James Nobles, Jessica Wheeler, Kirsty Dunleavy-Harris, Richard Holmes, Alan Inman-Ward, Alexandra Potts, Jennifer Hall, Sabi Redwood, Russell Jago, Charlie Foster

**Affiliations:** 1 The National Institute for Health Research Applied Research Collaboration West (NIHR ARC West) at University Hospitals Bristol and Weston National Health Service Foundation Trust, Bristol, UK; 2 Population Health Sciences, Bristol Medical School, University of Bristol, Bristol, UK; 3 Active Gloucestershire, Gloucester, UK; 4 SportEngland, London, UK; 5 Knowledge Change Action, Dursley, UK; 6 Institute for Sport, Physical Activity and Leisure, Leeds Beckett University, Leeds, UK; 7 Bradford Institute for Health Research, Bradford Teaching Hospitals National Health Service Foundation Trust, Bradford Royal Infirmary, Bradford, UK; 8 Faculties of Life Sciences and Health Studies, University of Bradford, Bradford, UK; 9 Centre for Exercise, Nutrition and Health Sciences, School for Policy Studies, University of Bristol, Bristol, UK

**Keywords:** Complexity, Systems science, Evaluation, Systems approach, Complex adaptive systems, Public health

## Abstract

**Background:**

Systems approaches are currently being advocated and implemented to address complex challenges in Public Health. These approaches work by bringing multi-sectoral stakeholders together to develop a collective understanding of the system, and then to identify places where they can leverage change across the system. Systems approaches are unpredictable, where cause-and-effect cannot always be disentangled, and unintended consequences – positive and negative – frequently arise. Evaluating such approaches is difficult and new methods are warranted.

**Methods:**

Ripple Effects Mapping (REM) is a qualitative method which can capture the wider impacts, and adaptive nature, of a systems approach. Using a case study example from the evaluation of a physical activity-orientated systems approach in Gloucestershire, we: a) introduce the *adapted* REM method; b) describe how REM was applied in the example; c) explain how REM outputs were analysed; d) provide examples of how REM outputs were used; and e) describe the strengths, limitations, and future uses of REM based on our reflections.

**Results:**

Ripple Effects Mapping is a participatory method that requires the active input of programme stakeholders in data gathering workshops. It produces visual outputs (i.e., maps) of the programme activities and impacts, which are mapped along a timeline to understand the temporal dimension of systems change efforts. The REM outputs from our example were created over several iterations, with data collected every 3–4 months, to build a picture of activities and impacts that have continued or ceased. Workshops took place both in person and online. An inductive content analysis was undertaken to describe and quantify the patterns within the REM outputs. Detailed guidance related to the preparation, delivery, and analysis of REM are included in this paper.

**Conclusion:**

REM may help to advance our understanding and evaluation of complex systems approaches, especially within the field of Public Health. We therefore invite other researchers, practitioners and policymakers to use REM and continuously evolve the method to enhance its application and practical utility.

**Supplementary Information:**

The online version contains supplementary material available at 10.1186/s12874-022-01570-4.

## Background

The term “complexity” is increasingly used within the field of Public Health. Complexity can describe a problem, an intervention, and / or the context in which an intervention is placed [1, 2]. Adding to this, there is often interplay between the three, whereby a complex intervention can be implemented in a complex system in response to a highly complex problem [1, 3]. Such degrees of complexity, and the inherent uncertainty, can make the evaluation of such phenomena exceptionally challenging. Public health issues such as physical inactivity and the prevalence of obesity are now regarded as the outcome of complex adaptive systems, meaning that a myriad of factors interact with one and other to cause these issues to occur and evolve over time [2, 4–8].

In response to these complex issues, systems approaches are widely being advocated and implemented [2, 4–11]. Systems approaches require the input and expertise from stakeholders working across various sectors and the community, to develop a shared understanding about the complexity of a problem and the surrounding context, and in turn, disrupt the system to change how it functions [12, 13]. These approaches often work in unpredictable ways, where cause-and-effect cannot always be disentangled, and where unintended consequences – positive and negative – frequently arise [8, 14]. They are somewhat impossible for stakeholders to control, and instead, proponents are encouraged to be agile; working with the system and adapting their approach accordingly – what Donella Meadows refers to as “dancing with systems” [15]. However, whilst the implementation of systems approaches has gained traction, the methods and mechanisms to study them are limited [5, 6, 8, 16–20].

Evidencing this is the recently published work by the School for Public Health Research (SPHR) [16, 17]. Egan and colleagues [5, 16, 17] summarised the methods which have been applied previously that take a systems perspective, identifying six: 1) applying systems thinking to qualitative inquiry; 2) systems mapping to understand the factors which may influence an outcome and / or an intervention; 3) network analysis to determine the relationships between people involved in the system; 4) systems dynamics modelling to examine how the system may respond over time to the hypothetical introduction of a intervention; 5) agent based modelling which simulate the response of a group of agents to the introduction of an intervention; and 6) uncategorised ‘systems friendly’ approaches. Similarly, the UK Medical Research Council (MRC) have provided guidance on how to evaluate *complex interventions* [1, 21–24], and how to evaluate complex interventions within complex social systems [1, 3, 21]. The methods proposed in these MRC documents are largely akin to those of the SPHR guidance. On the whole, these methods focus on either the relationships between stakeholders in a system, or the prospective modelling of how the system works or may respond to a hypothetical intervention. Whilst these guidelines offer a useful starting point for planning how to evaluate an intervention using a systems perspective, both documents (SPHR and MRC) call for new and innovative evaluation approaches [1, 5, 16, 17, 21].

**Fig. 1:**
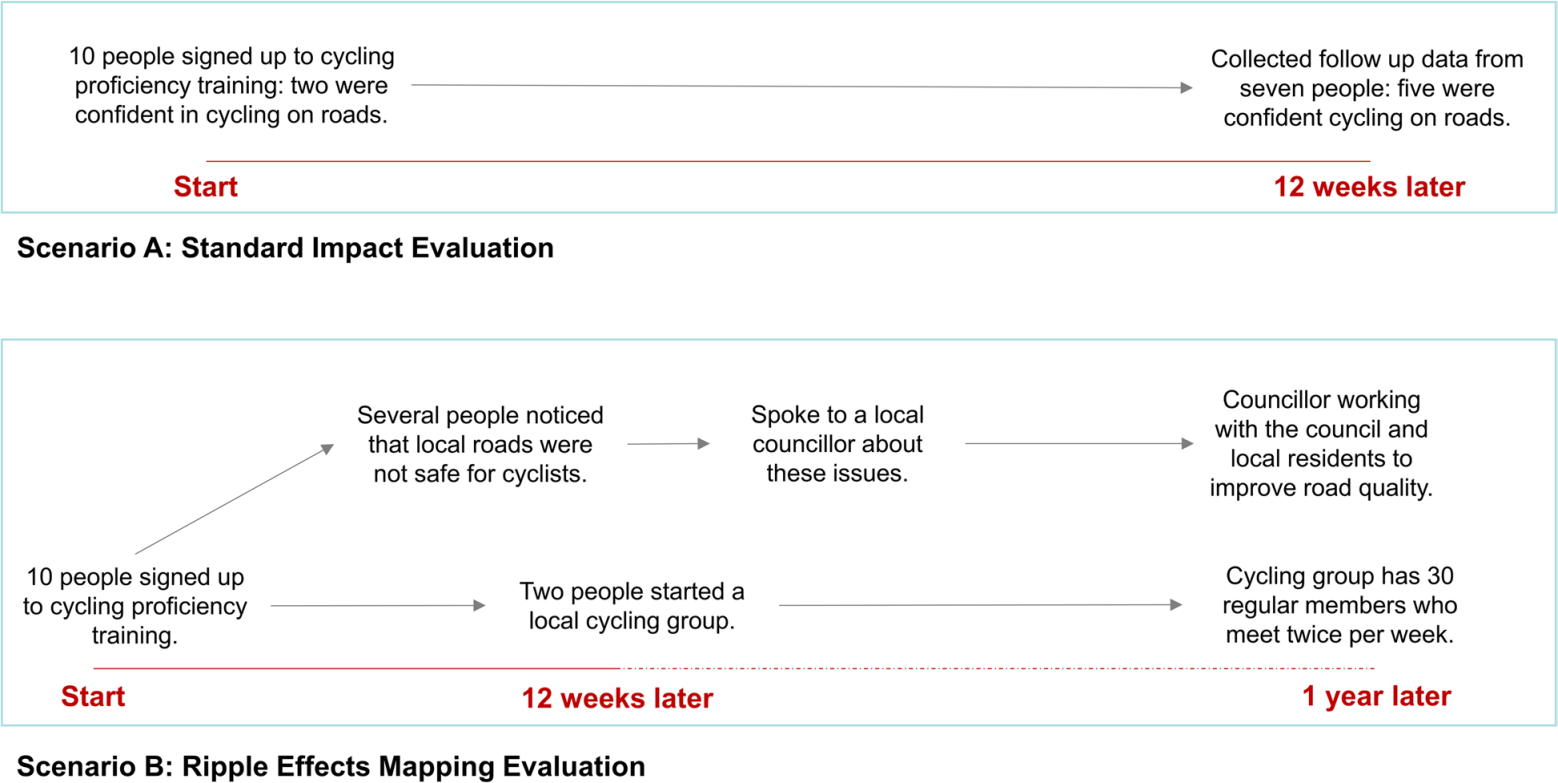
Hypothetical scenario using Standard- and REM- Evaluation

Ripple Effects Mapping (REM) is a method that can be used to better understand the dynamic nature and wider impacts of an intervention [25]. Unlike traditional evaluation designs that orientate themselves around studying attribution (e.g., Randomised Controlled Trials), REM is concerned with the study of contribution; how may an intervention, action or policy contribute towards changing an outcome or a system? It takes the form of a participatory qualitative impact evaluation, involving stakeholders who are engaged in, or are affected by, the intervention (e.g., delivery staff, commissioners, intervention designers etc.…). Stakeholders take part in a series of workshops to visualise the impacts of the intervention, and how these impacts may go beyond those which the intervention was designed to achieve [25]. For example, Fig. 1 demonstrates that a simple intervention such as cycling proficiency training may lead to impacts that go beyond increasing an individual’s confidence in their ability to cycle. These wider outcomes can demonstrate the additional value of an intervention. It can also highlight how an intervention may adapt in response to the system that it is situated within (or the context within the system), which often leads to unintended consequences, both positive and negative.

Ripple Effects Mapping has been used previously in North America to evaluate community-based interventions [25], some of which focus on the prevention of childhood obesity [26–28]. In their book, “A Field Guide to Ripple Effects Mapping”, Chazdon et al. [25] explain its underlying principles and present several approaches to applying REM (Table 1). REM is underpinned by four key principles, first, that it draws upon the concept of appreciative inquiry [29], second, that it takes a participatory approach, third, that it uses interactive group interviewing and reflection, and fourth, that it uses mind mapping to visualise the impacts. Given the increasing focus being placed on complex adaptive systems and systems approaches, in this paper we explain how REM has been adapted to help evaluate a systems approach to physical activity in Gloucestershire (a large county in South-West England). Based on our experience of using the method, we believe that REM will be useful in helping researchers and practitioners to explore the non-linear, unpredictable and dynamic aspects of systems approaches, whilst accounting for the emergent and adaptive nature of the complex system in which they are positioned.

**Table 1:** Underpinning principles and variations to REM

**Underpinning principles**	
1. Appreciative inquiry	Appreciative inquiry is an approach to creating generative knowledge, whereby stakeholders come together to reflect upon an issue or intervention, and to collectively think through what the future could look like – helping to establish energy and momentum amongst a group. Four phases of appreciative inquiry are often referred to: discovering, dreaming, designing, and delivery/destiny. REM predominantly focuses on the discovery phase.
2. Participatory approach	Stakeholders are seen as a core, active part of the evaluation rather than a unit of inquiry or recipient of an evaluation report.
3. Group interviewing and reflection	Data is gathered via participatory and interactive methods. This often includes stakeholders working together to create a shared understanding of what happened within an intervention. This takes the form of peer semi-structured interviews or focus groups. Reflecting on these conversations can also stimulate new ways of working between stakeholders involved in the REM.
4. Mind mapping	The resultant discussion between stakeholders is captured through diagrammatic processes, akin to that of a mind map, whereby the relationships between concepts are captured and organised in a hierarchical manner.
**Variations in REM**	
1. Web-mapping	Use a predetermined framework or theory to map short-, medium- and long-term impacts against. Recommends the use of the Community Capitals Framework.
2. In-depth rippling	The group focus on their perceived most important and impactful chains of events. A framework is not used to guide the group discussion but may be used to facilitate the analysis of the output.
3. Theming and rippling	The group collect impacts from all participants initially, and then generate themes from these impacts within the workshop. The wider impacts, or ripples, are then examined after themes are generated.

Adapted from Chazdon et al. [25]

This paper uses a case study example from an evaluation of a physical activity- orientated systems approach in Gloucestershire, to: a) introduce the *adapted* REM method; b) describe how REM was applied in the case study; c) explain how REM outputs were analysed; d) provide examples for how the REM outputs can be used; and e) describe the strengths, limitations, and future uses of REM based upon our reflections. Whilst we draw on a systems approach to *physical activity* in this paper, the method is transferable to other topics and fields that are entangled in complex adaptive systems.

## Methods

### Case study: we can move

We draw on the evaluation of We Can Move (WCM) in Gloucestershire, England [30] to provide an example for how REM has been applied. We Can Move brought together multiple organisations and sectors from across the county (e.g., local government authorities, NHS trusts, clinical commissioning groups, voluntary and community sector organisations, communities, and the public) to increase the opportunities for the local population to increase physical activity. It achieved this by adopting a systems approach. A core aim of WCM was to influence key organisations and leaders to mobilise assets across Gloucestershire in order to re-design and influence how the system works (that which impacts population physical activity).. The programme was co-ordinated and facilitated by Active Gloucestershire [31]. The National Institute for Health Research Applied Research Collaboration West (NIHR ARC West) was commissioned to evaluate WCM between April 2019 and April 2021. Ethical approval for this evaluation was granted by the Faculty of Health Sciences, University of Bristol (Ref. 91145).

### Ripple effects mapping and the wider evaluation

The lead researcher (JN) was embedded within Active Gloucestershire for 1 day per week, which enabled them to develop a thorough understanding of WCM and its intended aims [32]. It was through this immersive process and conversations with the programme team that REM was identified as a potentially useful and feasible method. REM was initially piloted in one specific project, before being applied to the wider WCM programme in December 2019. It is important to note that REM was one method situated within the larger evaluation of WCM.

### Ripple effects mapping

#### Preparation

Ripple Effects Mapping is a participatory method and data are collected through stakeholder workshops. The list below provides an overview of the preparatory work that was required. Further details are available in Online Supplement I.

- Planning the content of the REM workshop
- Deciding on a preferred format (face-to-face, online, or blended)
- Planning the logistical aspects of the workshop
- Planning for additional data collection
- On the day preparation activities

#### Stakeholder recruitment

Chazdon et al. [25] recommend that direct (e.g., implementation staff and participant) and indirect (i.e., those influenced as a by-product of the intervention) stakeholders are recruited. However, given that the core implementation team involved almost 20 people, a pragmatic decision was taken to deliver the initial workshop solely for this group. The group included employees of Active Gloucestershire (the organisation facilitating WCM) and key collaborators (e.g., local government authority and clinical commissioning groups). Further workshops were run separately for specific projects and wider stakeholders were invited to attend these. For example, a separate REM session was delivered with a group of community members who were integral to one specific project within WCM.

#### The initial ripple effects mapping workshop

This section is presented in two parts to describe what happened a) *during* the initial REM workshop and b) *following* the workshop.

##### During the initial workshop

The initial workshop was delivered over 2.5 h. The majority of time was allocated to mapping the WCM impacts. Two researchers were present, one facilitated the workshop and the other made observational notes. See Online Supplement I for further information.

*Presentation (20 min):* The background to REM, the rationale for its use, and an example of an REM output were presented to the group.

*Outline the REM process (10 min):* The facilitator presented an overview of the process for the REM workshop.

*REM activity (Two hours):* Participants divided into smaller, self-selected groups. Groups typically included three to five people, all of whom were familiar with the particular project / area of work being discussed. All participants worked on more than one project. There was sufficient time within the two-hour activity for each sub-group to work on two to three REM outputs, with each output corresponding to the respective project / area of work. The facilitator guided the group through the two-hour activity.

The first 10–15 min was allotted to the *team-based discussions*, underpinned by several of the appreciative inquiry principles as suggested by Chazdon et al. [25]. The purpose of these discussions was to discover what participants consider to be successful within WCM, or their best experiences, as part of WCM. They were encouraged to think about the relationships between stakeholders in the system, as well as their own projects and areas of work. They were then asked to think about what made these achievements or impacts possible, and whether these achievements came about in an expected or unexpected manner. Group members had these conversations in pairs. This activity was a gentle introduction to thinking about the ripple effects of their work, and the people and organisations that they may have had an impact upon. Data were not formally recorded at this point.

Each sub-group was provided with a large sheet of paper with a timeline drawn on it for the second stage of the workshop (*mapping the impacts, 90 min approx.*). The timeline is a notable addition to the approach of Chazdon et al. [25] as it allows the evaluation to understand the length of time required for certain impacts to arise (Online Supplement II). Here, timelines spanned from April 2018 (when WCM began) to December 2019 (when the first workshop was delivered). They were asked to reflect upon their work throughout that time, and to note key activities or actions accordingly against the timeline. They were then asked to think about the impacts that occurred following on from these activities or actions. Arrows were drawn between activities and impact(s) to illustrate the “ripple effect.” One participant acted as a scribe to visualise the REM output.

**Fig. 2:**
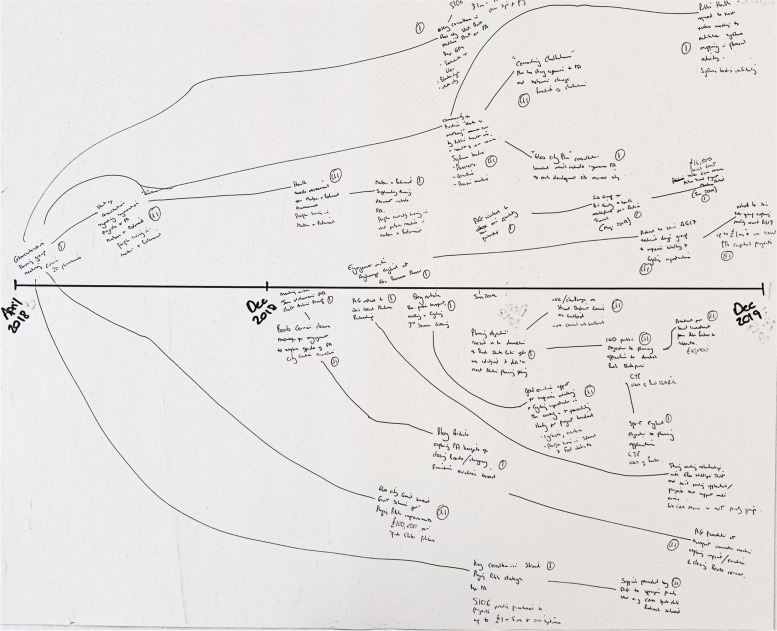
Example REM Output (Paper-based)

The third stage (*reflecting on the impacts*), which was delivered concurrently to stage two, involved participants further reflecting on their activities and impacts. As such, they were asked to consider the following: 1) who has been impacted upon (e.g., community members, organisations, system leaders); 2) how many people have been impacted; 3) whether there has been any financial implications associated (e.g., further funding generated); 4) if the impacts were intended or unintended; 5) what else may have contributed to these impacts; 6) whether their work links with wider WCM work or that of other organisations; and 7) if there are any recurring trends being observed across their REM output. Further detail was then added to the REM output. Throughout the workshop, each sub-group had time to work on two or three REM outputs. The facilitator moved between the groups to provide further assistance where required and to ask probing questions surrounding the REM output. Figure 2 provides an example REM output.

The fourth stage (*most and least significant changes, 10 min approx.*) involved participants identifying their most and least significant changes in the REM outputs. It was important to reflect on the least significant changes because these may denote actions and activities which required a lot of time and resource, but subsequently lead to little meaningful impact. When time permitted, participants were able to reflect upon why these activities lead to negligible impacts through discussion.

The last stage of the workshop (*group feedback and learning, 10 min approx.*) was for the group to reflect upon REM as a process. The group were asked questions such as: a) what have you learnt about your work from the REM outputs? b) who else could be involved in these REM workshops in the future? and c) having reflected upon your work and its associated impact, do you believe you are focusing on the right things?

##### Following the initial workshop

**Fig. 3:**
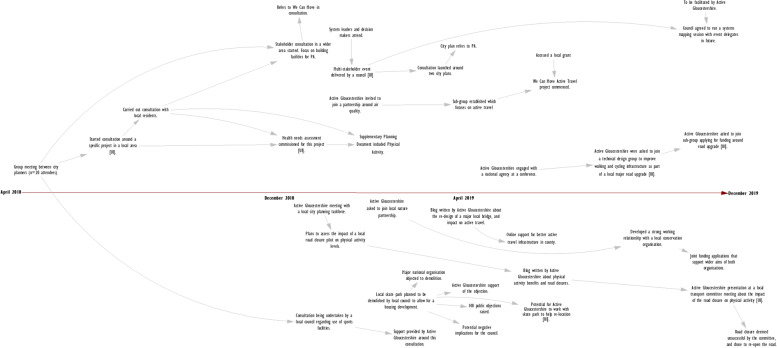
An Example REM Output (Digital)

The WCM core team created 12 REM outputs which covered various elements of the WCM programme. Immediately after the workshop, the researcher took a picture of all outputs to create a digital record. Each REM output was then systematically inputted into an online software package, Vensim (Fig. 3). The researcher contacted the WCM team if hand-written text was not legible to avoid misinterpretation. Outputs required between 30 and 60 min each to input into the Vensim software.

#### Follow up ripple effects mapping workshops

Chazdon et al. [25] suggests that REM is used to evaluate an intervention or project once its implementation is complete – with several published examples available [26–28]. However, we saw this to be problematic for two reasons. First, we believed that it might lead to an overly positive representation of programme activities and impacts. Second, we wanted to understand how systems approaches adapt in response to changes in the system. In our view, completing the REM output in a single workshop at the end of implementation would limit the potential for these adaptative and emergent properties of the system to be captured.

##### During the follow up workshops

We planned three follow up REM workshops in April 2020, July 2020 and November 2020. These workshops followed an abridged and simplified process compared to the initial REM workshop, which was adopted for all follow up workshops. As the groups became more familiar with the REM method, the time required to complete the follow up workshops reduced. Each workshop lasted between 60 and 90 min dependent on the volume of activity and impact that had occurred since the previous workshops.

Follow up workshops were completed online using Microsoft Teams as we were unable to meet in a face-to-face format due to COVID-19 mitigation measures. *Workshops were organised with people who were involved in the creation of the REM outputs* (i.e., the sub-groups) rather than the whole WCM team (as per the initial workshop). These sub-groups were able to update multiple REM outputs within the allotted time of the follow up sessions.

*Preparing for the online workshops:* The researcher familiarised themselves with the REM output (i.e., Vensim file) prior to the workshop and created a series of questions to ask the group about their REM output. The questions addressed three aims: 1) to seek further clarification on previous impacts and activities; 2) to update previous impacts and activities; and 3) to understand new impacts and activities that had not previously been discussed. If impacts and activities had ceased, then the researcher asked why this happened. These questions sought to avoid the REM outputs solely focusing on positive impacts and activities.

Approximately 2 weeks before the online workshop, the researcher contacted participants via email to explain what the workshop would consist of and to ask them to prepare for the workshop. They were also sent a copy of the electronic REM output to assist their preparation and to ensure that the output reflected the previous workshop discussion. This preparation was important in ensuring the online workshop was efficient.

*During the online follow-up workshop:* The researcher commenced the workshop by stating its aims and asked the group if they consented for the workshop to be recorded. Video-conferencing software (Microsoft Teams) allowed for the video, as well as the audio, to be recorded; this option was preferable to audio-only as the researcher could see which element of the REM output the discussion relates to. The researcher’s role was two-fold in the workshop. First, they guided the group through the series of questions related to their REM output, and ensured that all members had an opportunity to contribute to the discussion. Probing questions were also used to elicit further information. Second, the researcher captured the responses of the group and added this to the REM output on Vensim (screen sharing was enabled to allow the group to see the REM output being updated). The researcher did not need to capture all information given that the session was being recorded. Throughout the online workshops, the researcher continuously fed back their interpretation of what was said to the group to ensure the accuracy of the REM output.

##### Following the workshops

The researcher watched the workshop recording and refined the REM output in Vensim, and additional information was added to the output where required. On several instances, the researcher recontacted participants to seek additional clarification on the information included in the REM output. The detail within these outputs develops over time, as can be seen in the example in the results (Fig. 5) which was created over five iterations.

#### Analysis of the ripple effects mapping outputs

Chazdon et al. [25] recommend that a deductive content analysis is applied, underpinned by the Community Capitals Framework. However, we opted for a largely inductive content analysis to explore the patterns within the REM data rather than trying to code them against a predetermined framework. Our justification was that an inductive approach would help better understand the complexity of a systems approach. To do this, we used two sequential processes: 1) identification of “impact pathways”, and 2) a content analysis of the impact pathways. Analysis was undertaken after the final iterations of the REM outputs were completed.

**Fig. 4:**
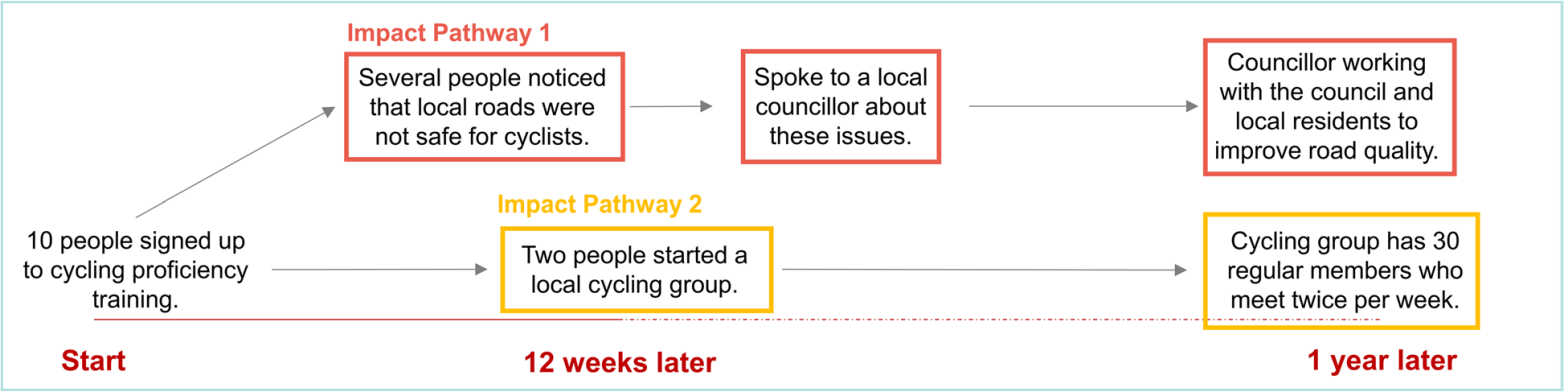
Impact Pathways

The research team immersed themselves in the data to identify impact pathways, i.e., chains of actions, activities and impacts within the REM output. Figure 4 provides a simple example of two impact pathways. Impact pathways predominantly served to facilitate the content analysis. We found that the process of applying a content analysis became easier having identified the impact pathways as they enable the REM data to be coded in the context of the impact pathway(s). The identification of impact pathways was completed in Microsoft PowerPoint, and we used different coloured boxes to demarcate the various pathways within the REM outputs. A PDF was created of the REM output with the finalised impact pathways which was imported into NVivo 12.

The data within the impact pathways were then systematically coded in NVivo 12 using content analysis [33]. All data were subject to coding, and we coded one impact pathway at a time. Where similar data were found in the output (e.g., a similar type of activity or impact), we applied a previous code – this enabled us to start building up a numerical, as well as descriptive, overview of the data. More than one code could be applied to a data extract. After coding three or four impact pathways, we began organising the codes into preliminary themes (i.e., clusters of codes which help to describe the phenomenon being observed). The data within subsequent impact pathways were coded against these preliminary themes; however, where data did not fit these themes, new codes were created.

Whilst much of the analysis was inductive, we had a set of specific questions that we aimed to answer. These included the estimated reach of the projects and the programme, the type of people and organisations involved in WCM, the length of time for impacts and activities to occur, and the financial implications of certain impacts and activities. As such, we created themes that related to these questions and coded data accordingly. This process allowed us to provide quantitative answers to these particular research questions / foci.

## Results

Throughout the course of the WCM evaluation (April 2019 – April 2021), we created 15 REM outputs by working with all members of the implementation team (*n* = 17) and eight members of the public (for one community-based project only). Seven of these outputs were subject to follow up workshops, of which one was updated four times, two were updated three times, two were updated twice, and two were updated once. Fourteen of the 18 follow up workshops were completed online (four follow up workshops were completed prior to the COVID-19 pandemic). Delivering REM workshops in an online format is more resource intensive, and so a practical decision was made to only follow up on the seven projects which had continued to be delivered throughout the COVID-19 pandemic. The results section will focus on one REM output for the purpose of this methodological paper.

### Presentation of the ripple effects mapping output

**Fig. 5:**
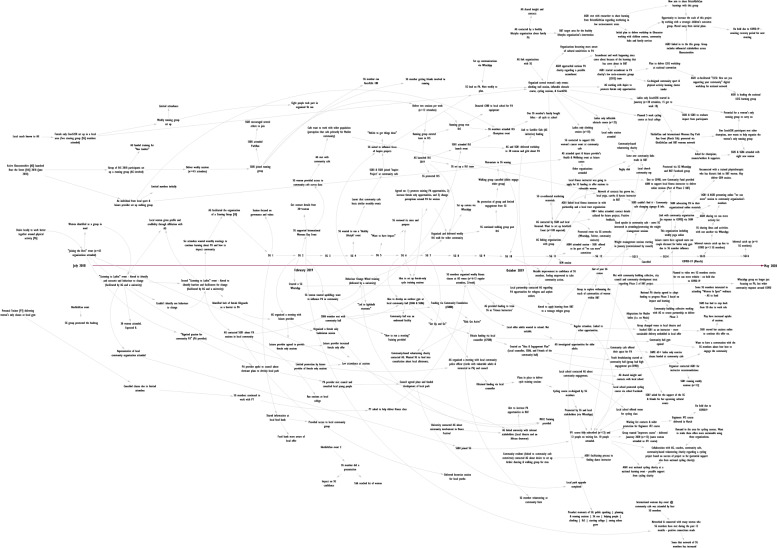
An Example Finalised REM Output (Digital)

Figure 5 illustrates the final anonymised REM output from a community-based project in Gloucester. The REM output was created over five iterations, which included four workshops with the project implementation team, and one workshop with eight stakeholders and community members who were involved in the project (who formed a project steering group). We mapped the activities and impacts between July 2018 and May 2020, but the project continued to run thereafter.

### Presentation of the ripple effects mapping impact pathways

**Fig. 6:**
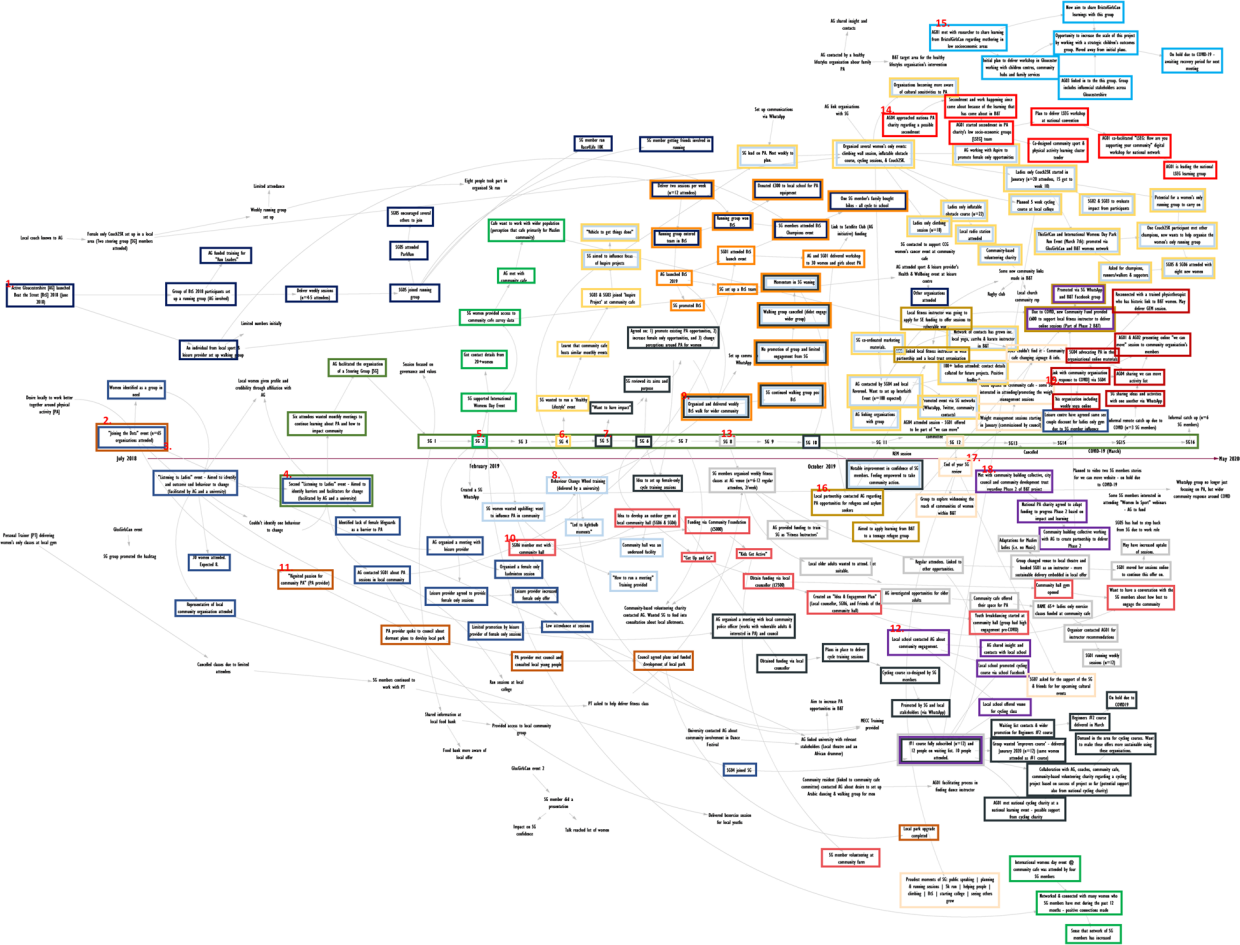
An Example of Impact Pathways on an REM Output

We identified 19 impact pathways within the example REM output (Fig. 6), and as evidenced, many pathways overlap. The primary purpose of the impact pathway is to facilitate content analysis. There were also some instances where data were not included within in an impact pathway as they were deemed superfluous. That said, all data were subject to content analysis, regardless of whether they were included in an impact pathway or not.

A secondary purpose of the impact pathway is to help bring the output to life given the complexity of the REM output as a whole. Taking Impact Pathway #7 as an example (Fig. 6), we can see that there was a desire amongst the community stakeholders to establish a women’s-only cycle training course. Through the impact pathway, we can see that these stakeholders met with local politicians to secure funding for the course, which in turn led to a course being co-designed by the stakeholder group. This cycling course was promoted locally, predominantly via word of mouth within the community, and was subsequently fully subscribed. A local school offered their outdoor playground as a space to deliver the course. This course was deemed successful by the stakeholders as it demonstrated a local demand for cycling amongst women in the community. Further courses were organised and conversations were held with a national cycling charity, local community organisations, and cycling coaches to create a sustainable delivery model for future cycle training courses. Illustrative pathways such as this can help external stakeholders (e.g., funders) understand the activities being implemented and some of the impacts that occurred thereafter.

This example highlights: a) the length of time required to establish a course such as this; b) the number of women engaged in the activity; c) the additional money secured to implement the training; d) the community assets that were involved; and e) the linking in with local and national organisations. By systematically appraising the impact pathways in this manner, we can then understand the wider impacts of work such as this, and how it evolves and adapts over time.

### Content analysis

We developed several key themes from this REM output (Fig. 6) to explain the types of impacts observed, the people and organisations involved, the mechanisms leading to these impacts, and the length of time required to generate these impacts. Regarding the impacts identified, these broadly spanned four themes: a) financial investment; b) stakeholder buy-in; c) self-organisation; and d) negative impacts. We identified that over 30 organisations were either involved in or affected by the project during the evaluation period, and almost 250 women were known to have participated in one or more of the initiatives being delivered. We grouped the mechanisms leading to impact into three themes: a) the role of the steering group; b) the role of Active Gloucestershire; and c) enabling factors which contributed to the observed impacts. Lastly, we identified three themes when estimating the timelines around impact, the time taken to: a) plan and deliver discrete initiatives; b) engage stakeholders; and c) change local infrastructure. Analysis of the most- and least- significant impacts could also be presented here (in the WCM evaluation, we did not systematically record this information as it was viewed as a reflective activity rather than an evaluative one). As the purpose of this paper is to illustrate the method, further information pertaining to these themes are not included here.

### Wider application of the ripple effects mapping findings

**Fig. 7:**
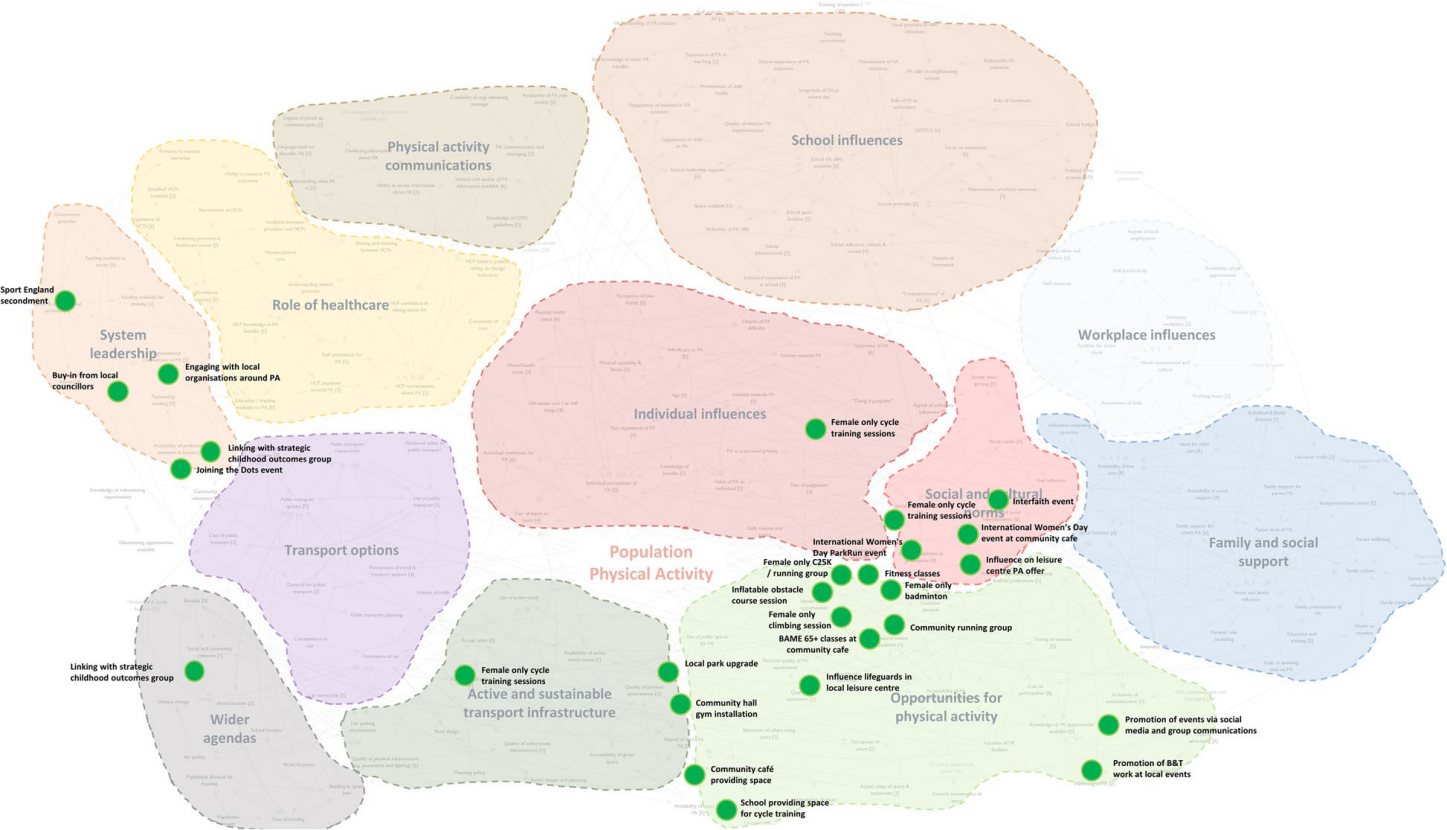
Actions overlaid on a systems map

As part of the wider evaluation of WCM, we worked with over 100 stakeholders to develop a systems map of the factors perceived to influence physical activity levels in Gloucestershire. The map includes 11 themes, from local transport options, to social and cultural norms, to school and workplace influences. Using the information obtained from the example project, most actions targeted the “opportunities for physical activity” (Fig. 7). The purpose of this activity was to bring together complementary evaluation methods, the results from which can help stakeholders to reflect upon their approach to date within a project or programme. The triangulation of methods and data sources facilitated the comprehensive evaluation of WCM.

## Discussion

### Summary

In this paper, we introduce and describe an adapted version of REM which may be useful for researchers, evaluators and practitioners who are studying complex interventions (inclusive of diet- and physical activity-related approaches) being delivered within complex systems. Our adaptations to the REM method (Online supplement II) mean that it can be undertaken alongside the implementation of an intervention, such as WCM in Gloucestershire, to identify the wider intended and unintended impacts. It has also been a beneficial tool to understand some of the mechanisms – and chains of events – that might explain why an intervention produces the impact(s) that it does. Moreover, in light of the interdependency between many complex issues – e.g., obesity, undernutrition and climate change [11] – REM provides an opportunity to identify double or triple duty actions; that is, where a single action impacts upon more than one issue simultaneously [1, 11]. Given the perceived utility of the method, this paper provides a systematic process for carrying out REM.

### Advancing the method

We advanced the methods of Chazdon et al. [25] in several ways, as outlined in Online Supplement II. These adaptations included the: 1) addition of a temporal dimension through a timeline; 2) identification of impact pathways; 3) emphasis on follow up workshops; 4) ability to deliver REM online; and 5) viewing REM as a prospective as well as a retrospective method. The latter is particularly noteworthy. The REM workshops, as described, encouraged participants to map out the activities and impacts that they anticipated will occur in the coming months. These anticipated activities and impacts can then be revisited in future REM workshops. This is important for two reasons. First, it enables evaluators and involved parties to examine the alignment between the intended impact pathways and the actual manifest impact pathways. When working within complex systems, interventions cause the system to change and adapt, often in unpredictable ways. Other contextual factors may also cause the system to adapt (e.g., wider policy implementation, changes in local and national governments, infectious disease outbreaks etc..). The REM output can be used to illustrate changes to the system, and therefore help to explain how and why the trajectory of the intervention differed from that which was planned. Second, the prospective mapping further embeds appreciative inquiry into the method. In the method described by Chazdon et al. [25], REM is predominantly viewed as a means of *discovering* what has occurred within a programme. Our prospective mapping encouraged participants to envision the future of the intervention and its associated impacts – aligned with the *dreaming* phase of appreciative inquiry [29]. This is particularly important when implementing a systems approach as it enables stakeholders, who may represent different sectors of the system, to clarify and align their thinking around the intervention. Doing so may help to create momentum towards the desired future state [25].

### Strengths of the method

We envisage that the REM method can be carried out by researchers, practitioners, and / or policymakers. Whilst the process of completing the *REM workshops* is relatively straightforward, is not resource intensive, and can be done within reasonably short timeframes, those wishing to apply the method should ensure that they have capacity and capability to analyse the REM outputs. Our experience would suggest that it may be important to have an independent person present in the REM workshops (either facilitating or observing), who can then carry out the *analysis* of the data objectively and based only upon the information present in the output.

REM may also act as an intervention in itself. Because REM brings together a range of stakeholders involved in, or influenced by, an intervention, we found that the workshops offered an opportunity to pause and critically reflect on their progress to date. This reflection can subsequently inform the future direction of their work – aligning with the ethos of developmental evaluation [14]. Similarly, when intervening in a complex system in ways that does not always lead to tangible change, REM is able to demonstrate softer impacts such as mindset shifts in organisational leaders, the setting up of infrastructure to support a systems approach, or the development of a stakeholder network [34]. Just as systems mapping has demonstrated that it can help stakeholders to see the complexity of a problem [35], we hypothesise the REM will enable stakeholders to document the impacts of their efforts and see how they have contributed or are contributing to a systems approach.

Another strength of the REM outputs was that they helped to identify key stakeholders for interviewing as part of the WCM evaluation. As Egan et al. [17], suggest, a systems evaluation should – wherever possible – gather the perspectives of those who sit on the periphery of an intervention. The REM output associated with the community-based initiative in WCM (Figs. 5 and 6) evidenced that over 30 organisations had been involved, therefore creating a sample from which we could interview. We used the REM outputs in the interviews to gather their perspectives on the aspect of the intervention that they had been involved in or affected by. Here, REM outputs serve as an elicitation tool rather than an interview guide (akin to a graphic elicitation techniques [33]). It was outside of the scope of this paper to include interview findings, but it is important to note that interviews can bolster the REM output, and help provide further context and explanation around the impact pathways.

### Limitations of the method

We also note several limitations of REM in this adapted form. First, evaluators must carefully attend to the activities and impacts that may otherwise get overshadowed by the major successes of an intervention. Ensuring that follow up REM workshops are planned into the evaluation are important, and facilitators should allocate sufficient time to update all impacts and activities that were mapped previously. Second, mapping all the ripple effects of a systems approach is unlikely to be feasible. It should not be the intention of the method to try and capture all associated activities and impacts, but rather to capture a range of impacts to demonstrate a balanced view of the work being undertaken or fall within pre-defined boundaries of the evaluation. Last, from our experience, REM seemed to work better for projects that are open-ended and highly adaptable rather than rigidly and / or prescriptively defined (i.e., transactional projects). Whilst we did see some value in applying the method to transactional projects, and indeed some of these projects do adapt due to contextual factors, REM was more valuable for evaluating the aspects of WCM that evolved over time, often born out of conversations and relationships between stakeholders. Systematic evaluation of REM as a method, which would gather the perspectives of participants from various stakeholder groups, is warranted in the future to better understand its strengths and limitations.

### Reflections and practical considerations for ripple effects mapping

Having utilised the REM method for over a year within the WCM evaluation, we have several reflections on how the process could further be refined. Table 2 provides several practical considerations for others looking to apply REM. We encourage other researchers to use this method, and having done so, to contribute to its methodological advancement over time.

**Table 2:** Considerations for REM

Practical considerations when using Ripple Effects Mapping	
**Preparation**	• Spend time working with project staff / implementers to understand the logic by which the intervention is anticipated to work. Seek to understand the broader context that the project is situated within. • Carefully consider the probing questions for the workshop. These probes will help to gather data that is pertinent to the research question, as well as providing structure for workshop participants. • Consider who would be most appropriate to facilitate the workshops and to analyse the data; this could be an independent research team, an embedded research team, or members of the implementation team. There are strengths and limitations to each of these approaches. • It may, or may not, be desirable to have a formal presentation at the beginning of the workshop. Researchers should work with implementers to determine what the preferred / most accessible format is likely to be.
**Recruitment**	• Work with implementers to invite a broad range of stakeholders (community members, differing sectors, organisations, and levels of seniority) who have been involved in / or affected by the project. • Use REM outputs to help identify additional wider stakeholders and work with implementers to invite them to future sessions. • Researchers / workshop facilitators may wish to speak directly with prospective participants to familiarise them with the method prior to their workshop attendance. • If wider stakeholders are not able to attend, consider using a semi-structured interview to ascertain similar information. This information can then be added to the developing REM output.
**Initial REM workshop**	• The role of the researcher in the mapping activity is to guide the conversation and to uncover further activities and impacts. This will be a similar role to that of facilitating a focus group discussion. • It would be useful to have several researchers present in the workshop to facilitate the group-based discussions (i.e., a facilitator per sub-group), especially at the beginning of the mapping when more queries are likely. Facilitators could also be members of the implementation team (e.g., Active Gloucestershire) if provided with sufficient training. • The initial session could also be completed in an online format dependent on researcher and group preferences or circumstances. If using a face-to-face format, Dictaphones could be used to capture some of the conversation being had whilst stakeholders are creating and discussing the REM output.
**Follow up REM workshops**	• Identify a mechanism for workshop attendees to record activities and impacts between REM sessions. These notes can then be drawn upon in the REM workshops and ensures key information is not overlooked / forgotten. • Re-familiarise with the REM output and create a set of questions to elicit further information from participants in the follow up workshop(s). • Allow the previous REM output to form the basis of the follow up workshop. Seek to update and expand upon this. Ask for updates across all aspects of the REM output to understand which aspects have led to further ripple effects and those which have not. • Ask to record the workshop if using video-conferencing software (e.g., Microsoft Teams, Zoom, Skype etc.…). This will enable the researcher to revisit the video recording to bolster the REM outputs.
**Analysis**	• When identifying impact pathways, remember that their purpose is to assist the subsequent analysis. It is likely that the identified pathways may differ between researchers. • A deductive approach may be useful if a particular theoretical or conceptual framework would help to answer the research questions.

## Conclusions

In this paper, we describe the adapted version of the REM method. We highlighted the circumstances in which REM may be applicable, alongside pointing out its strengths and limitations. We believe that REM could hold considerable potential as a method to advance our understanding and evaluation of complex systems approaches, especially within the field of Public Health. We therefore invite other researchers, practitioners and policymakers to use REM and to continuously evolve the method to enhance its application and practical utility.

## Supplementary Information

**Additional file 1: Online supplement I:** Further information related to the process of Ripple Effects Mapping.

**Additional file 2: Online supplement II.** Modifications made to Ripple Effects Mapping.

## Data Availability

All materials related to the REM method are provided in the manuscript and online supplementary materials.

## Abbreviations

REM: Ripple Effects Mapping
WCM: We Can Move
PA: Physical Activity
NHS: National Health Service
