# Supplementary material for: Ripple effects mapping: capturing the wider impacts of systems change efforts in public health
Source: BMC Med Res Methodol. 2022 Mar 18;22:72. doi: 10.1186/s12874-022-01570-4 (PMC8930282; doi:10.1186/s12874-022-01570-4)
Supplement: Supplementary file 1 — Additional file 1: Online supplement I: Further information related to the process of Ripple Effects Mapping. [file 12874_2022_1570_MOESM1_ESM.docx]

**Online supplement I**: **Further information related to the process of Ripple Effects Mapping**

This supplement aims to provide more detailed information related to the process of Ripple Effects Mapping. The information presented here corresponds with each of the sections in the main paper.

**Preparation**

Preparatory work included the following:

- *Planning the content of the REM workshop*: The lead researcher created a presentation to provide a background to REM, to justify this choice of method, to give an illustrative example of the method (using the pilot project), and to explain the process that would be followed in the workshop.
- *Planning the logistical aspects of the workshop*: It was important to find a setting that had sufficient room to carry out the REM workshop, including tables for group-based activities. The chosen location was in the same building as the Active Gloucestershire staff to reduce travel burden. The research team also needed to source materials for the workshop, including: large pieces of paper (e.g. A0), coloured pens, post-it notes, and a projector.
- *Planning for additional data collection*: The research team also asked a colleague (independent of the project), who was familiar with observational methods, to attend the workshop. Their role was to collect feedback and observations from the workshop that would allow further refinement of the method thereafter. This researcher was briefed before the workshop.
- *On the day*: The researcher organised the room to promote group conversations, and also drew a timeline onto each piece of A0 paper.

**The initial Ripple Effects Mapping workshop**

*During the workshop*

*Presentation (20 minutes):* The background to REM, the rationale for its use, and an example of an REM output were presented to the group. The facilitator tailored the presentation content so that the examples and scenarios were relatable and salient. For example, when presenting an REM output to the group, the facilitator used that which arose from the piloting of the REM method in one WCM project. It was anticipated that this tailored content would help strengthen the rationale for using REM in the context of the WCM programme.

*Outline the REM process (10 minutes):* The facilitator presented a detailed overview of the process that would be followed throughout the remainder of the REM workshop. The process was laid out across five stages: 1) team-based conversations; 2) mapping the impacts; 3) reflecting on the impacts; 4) identifying most and least significant changes; and 5) group feedback and learning. The entire process was described before the workshop activities commenced. This was to ensure that the group understood the sequencing of the activities and how the activities build upon one another.

All information related to the activity component of the workshop is retained in the main article.
