# Supplementary material for: Ripple effects mapping: capturing the wider impacts of systems change efforts in public health
Source: BMC Med Res Methodol. 2022 Mar 18;22:72. doi: 10.1186/s12874-022-01570-4 (PMC8930282; doi:10.1186/s12874-022-01570-4)
Supplement: Supplementary file 2 — Additional file 2: Online supplement II. Modifications made to Ripple Effects Mapping. [file 12874_2022_1570_MOESM2_ESM.docx]

**Online supplement II: Modifications made to Ripple Effects Mapping**

This supplement presents the original method of In-depth Rippling as proposed by Chazden *et al*. (2017) and then illustrates the adaptations made by Nobles *et al*. (as presented in the main paper).

| **Original REM Method (Chazden *et al.,* 2017)** | **Adapted REM Method (Nobles *et al.)*** |
| --- | --- |
| **Purpose** |  |
| To retrospectively document the results of programme efforts within complex, real-life settings by visually mapping the intended and unintended changes (pg. 1-2). | To visually map the past, present and anticipated wider impacts (intended and unintended) and activities that are associated with a complex, real-life programme. |
| **Principles** |  |
| 1. *Appreciative inquiry*: Primary focus of REM here is to focus on the Discovery phase (i.e. all participants understand what has happened as a result of the programme). Discussions in the workshop may move towards other aspects of appreciative inquiry, including Dream, Design and Destiny phases. | 1. *Appreciative inquiry*: Adapted method focuses on both the Discovery and Dreaming phases of appreciative inquiry. Participants are encouraged to collectively understand the impacts of the programme (i.e. Discovery) and to highlight what they anticipate will happen in the future (i.e. Dream). These conversations are anticipated to influence discussions around the future design of the programme. |
| 1. *Participatory approach*: See’s programme stakeholders as active participants in the evaluation process rather than passive recipients of information about the evaluation. | 1. *Participatory approach*: No substantial changes. |
| 1. *Group interviewing and reflection*: Peer-to-peer semi-structured interviews are proposed in the first part of the workshop to collect information about the positive impacts of their work (underpinned by appreciative inquiry). Information is recorded. In the second part of the workshop, group interviewing and reflection is used to gather information pertaining to the wider programme impacts (alongside the visual mapping). | 1. *Group interviewing and reflection*: No substantial changes. |
| 1. *Mind mapping*: Mind mapping is used to visually depict the chain of effects resulting from a programme. | 1. *Mind mapping*: The main adaptation is the inclusion of a timeline. Participants are asked to map their activities and associated impacts against this timeline. The links between activities and impacts are illustrated as per the original method. |
| **REM workshop** |  |
| 1. *Introduction and overview*: Participants informed of the workshop aims and introduced to the REM method. | 1. *Introduction and overview:* No substantial changes. |
| 1. *Team or group-based discussions*: Peer interviews, with questions underpinned by appreciative inquiry, are carried out. Conversations mainly focus on the successes of the programme, what people are proud of, and what they have learned. 2. *Mapping*: People are asked to share their stories from the peer interviews (step 2), and the facilitator and other workshop participants, ask further questions to ascertain further information. These details, and the ripples associated with an activity or impact, are drawn onto a sheet of butchers / flip chart paper. Work around the group until all stories have been captured. This requires the greatest amount of time in the workshop. 3. *Reflecting on the impacts*: This final section of the workshop asks the participants to reflect on the REM output they have created, and ask several questions: 1) what do they see as the most significant change? 2) what is most interesting about the map? 3) how can the map be used to make a difference? and 4) what can they all do next? (moving towards action). | 1. *Team or group-based conversations*: No substantial changes. This part of the workshop is predominantly viewed as a warm-up to the main mapping activities. 2. *Mapping*: Participants are asked to group themselves in line with the projects they work on or are involved in. Each group are given a piece of butchers / flip chart paper that has the timeline running along the centre. They are asked to reflect on their work during this time, and add notable activities and impacts to the paper. They are then asked to think about the impacts that occurred following on from these activities or actions. Arrows are drawn between activities and impact(s) to illustrate the “ripple effect”. 3. *Reflecting on the impacts*: This step is delivered concurrent to step 3. Once some of the initial activities, impacts, and “ripple effects” have been mapped out, a series of probing questions can be asked to get participants to add further detail to their outputs (probing questions are included in the main manuscript). Towards the end of the mapping, participants are then asked to reflect on the most and least significant changes. Participants can also be encouraged to map the activities and impacts that they anticipate will happen in the next three-to-six months (revisited in follow up workshops). A final set of questions are then asked to understand what participants have learnt from the workshop. Multiple outputs can be created within the allotted time. |
| *Logistics*: Suggest that all relevant stakeholders come together in a face-to-face workshop. Workshop to last approximately two hours. | *Logistics*: Initial session can be completed in a face-to-face or an online workshop. All relevant stakeholders invited to attend. Workshop to last approximately two hours. |
| *Facilitation style*: Chazden and colleagues (2017) suggest that facilitators have a role that is similar to that required in a focus group. | *Facilitation style*: No substantial changes unless the REM session is held online. In which case, the facilitator holds a dual role of a) managing the group as one would a focus group, and b) creating the REM output on the chosen software package. |
| **Follow up REM workshops** |  |
| No follow up REM workshops. | Follow up workshops were held every 3-4 months following the initial REM session. The purpose of these workshops was to reflect on the prospective mapping that occurred in the initial session (to determine if activities and impacts had unfolded as planned), and to add further impacts and activities to the REM output. Participants were asked to update on all aspects of the previous REM output (some of which would not have progressed as planned). Participants can also prospectively map the activities and impacts for the forthcoming 3-6 months. It was anticipated that the inclusion of follow up workshops would reduce recall bias and avoid the mapping activity being overly representative of positive impacts.  *Logistics*: Follow up workshops can be delivered in a face-to-face or online format. Should allow 60-90 minutes for workshop to be completed. |
| **Analysis** |  |
| The hand drawn REM outputs from the workshop are converted into a digital copy. Once the final outputs are agreed upon (may require further feedback from workshop participants), the digital output can be coded.  It is recommended that the Community Capitals Framework is used to then code the data in the outputs against. However, the authors state that other frameworks may be more appropriate. This approach is similar to a thematic (and/or content) analysis in qualitative research. Dual coders are recommended. | All REM outputs will be digitalised as per the original method.  The first step of analysis in the modified REM method is to identify the impact pathways. This helps the evaluator to understand the context in which the data is placed, and the pathways can also be used to help implementation staff to identify salient stories which may provide good representations or illustrations of their work. It is likely that these impact pathways will overlap substantially with each other.  The second stage of the analysis is to use a content analysis to code and organise the REM data into themes. A pre-determined framework was not used in this adapted model. Some of these themes could then be quantified. |
